# Clinical study of cerebral palsy in 408 children with periventricular leukomalacia

**DOI:** 10.3892/etm.2015.2222

**Published:** 2015-01-27

**Authors:** QING SHANG, CAI-YUN MA, NAN LV, ZHONG-LI LV, YI-BING YAN, ZHI-RONG WU, JING-JIE LI, JIA-LI DUAN, CHANG-LIAN ZHU

**Affiliations:** 1 Children’s Hospital of Zhengzhou, Zhengzhou, Henan 450053, P.R. China; 2 Beijing Children’s Hospital, Capital Medicine University, Beijing 100045, P.R. China; 3 Children’s Hospital of Liaocheng, Liaocheng, Shandong 252000, P.R. China; 4 Center for Brain Repair and Rehabilitation, Institute of Neuroscience and Physiology, The University of Gothenburg, Göteborg 40530, Sweden

**Keywords:** cerebral palsy, periventricular leukomalacia, high risk factor, comorbidities

## Abstract

This study aimed to investigate the high risk factors, cerebral palsy (CP) subtypes and comorbidities of periventricular leukomalacia (PVL). Based on treatment conditions at a specialist hospital, a cross-sectional clinical study and retrospective analysis of computed tomography and magnetic resonance imaging examinations was conducted to evaluate the risk factors, subtypes and comorbidities of CP in children with PVL. Among the 408 children with PVL, 8.58% were born with a weight of ≤1,500 g and 44.36% were born with a weight of ≥2,500 g. In addition, 36.76% of these children had a gestational age of ≤32 weeks and 37.75% had a gestational age of ≥37 weeks. The proportion of the children born with various high risk factors was 95.59%, including perinatal infections and hypoxia. Severe PVL was observed in preterm infants (63.41% with a gestational age of <28 weeks and 21.95% with a gestational age of 28–30 weeks) and low-birth weight infants, which were prone to quadriplegia (43.90%). The common comorbidities included visual and auditory disorders, epilepsy, mental retardation and language barriers. Visual and auditory disorders (26.96%) were the most common comorbidities. PVL was identified primarily in premature and low-birth weight infants. The degree of PVL was found to be negatively correlated with gestational age and birth weight. The degree of PVL in the full-term infants correlated with exposure to infections or hypoxia. Quadriplegia is common among the various subtypes of CP. Visual and hearing disorders are the most common comorbidities of CP; these comorbidities occurred most frequently with quadriplegia.

## Introduction

With the improvement of perinatal medicine and neonatal intensive care in recent years, the survival rate of low-birth weight infants, premature infants and infants with severe asphyxia has been significantly improved. Consequently, the incidence of neonatal brain injuries has increased; in particular, periventricular leukomalacia (PVL) has attracted increasing attention (1).

It has been proposed that PVL occurs due to perinatal inflammation and/or hypoxia-ischaemia induced by various factors during the perinatal period (2). Anatomical pathology has shown that the periventricular white matter of the watershed areas of the lateral ventricles exhibits oedema and coagulative necrosis with a macrophage response to form cysts following hypoxia and ischaemia of the brain. Areas of cystic necrosis can collapse to form scars resulting in gliosis. The periventricular white matter is extensively replaced with numerous cysts due to white matter deficiency in periventricular areas and the centrum semiovale. The ependyma between the softened area of the cyst and the ventricle is destroyed; the integrity of the cysts with the ventricle is also disrupted. The ventricle locally or passively expands to form jagged edges, exhibiting an irregular shape. Commonly observed in preterm infants and full-term children with suffocation, PVL is a significant manifestation of infant brain injuries. PVL is the main cause of infant mortality; patients who survive suffer from nervous system disorders and mental retardation later in life. The pathogenesis of this condition has been linked to perinatal inflammation and/or hypoxia-ischaemia. Ischaemia/reperfusion results in the formation of reactive oxygen species and reactive nitrogen species, causing cell death (3).

In addition to early mortality due to the poor prognosis of PVL, 50% of the sequelae from cerebral palsy (CP) are related to the manifestation of PVL in survivors (4). Studies have shown that PVL is an independent risk factor of CP (5,6). CP is characterised by a group of developmental processes involving movement and posture; as a result, activity is limited and this finding is attributed to non-progressive disturbances that occur in the developing foetal or infant brain (7). CP is a serious disorder in children and influences the survival and quality of life of infants. However, at present, no specific treatment is available to alleviate PVL; hence, the prevention of various risk factors associated with PVL is necessary.

Therefore, in the present study, the clinical data of 408 children with CP were retrospectively analysed by examining images of PVL in the Rehabilitation Centre of the Children’s Hospital of Zhengzhou (Zhengzhou, China). The correlations of the PVL degree, gestational age, low birth weight, asphyxia and other high-risk factors, as well as the formation, type and comorbidities of CP were analysed to provide an objective basis for the prevention of illness, clinical analysis and prognosis of children with CP. This study provides guidelines for perinatal prevention, the early clinical diagnosis of PVL, early intervention and the reduction of sequelae.

## Subjects and methods

### Subjects

Among the 2,700 children with CP that were subjected to rehabilitation therapy in the Rehabilitation Centre from August 2008 to August 2012, the following cases were observed: 43.57% spastic diplegia; 42.53% spastic quadriplegia; 5.39% spastic hemiplegia; 3.73% muscle hypotonia; and 2.49% involuntary movements. Approximately 89% of these patients exhibited abnormalities in the computed tomography (CT) or magnetic resonance imaging (MRI) of the head relevant to the ages of the children examined in the hospital. The common results of the CT or MRI were listed as follows: widened subarachnoid cavity; enlarged ventricles; mild brain atrophy; PVL; and low density. The CT/MRI results of the heads of 2,700 patients indicated that 408 children (15.11%) suffered from PVL including 301 males and 107 females. The ages of these 408 patients ranged between five months and 10 years with an average of 18.67±17.38 months. Among the 408 cases included, 330 were aged below two years, 57 were aged between two and four years, 13 were aged between four and six years, and 8 were aged more than six years (the symptoms of these children were mild and inappropriate for surgery). The gestational age was between 24 and 41.6 weeks, with an average of 34.67±4.62 weeks. The birth weight ranged between 900 and 5,500 g, with an average of 2,480.47±750.60 g, including 226 cases with a birth weight of <2,500 g. This study was conducted in accordance with the Declaration of Helsinki and with approval from the Ethics Committee of the Children’s Hospital of Zhengzhou. Written informed consent was obtained from the parents or guardians of the participants.

### PVL diagnosis and analysis

MRI has been demonstrated to be a valuable tool for monitoring the development and pathology of the preterm brain. In this retrospective study, some of the CT results were obtained from other hospitals prior to the children being admitted to the Children’s Hospital of Zhengzhou. In addition, the MRI examination exhibits high noise, and some children with CP exhibited sleep disorders. Although these children were anaesthetised, the MRI examination could not be completed. As a result, some children were subjected to CT scanning.

A 64 row spiral head CT scanner (GE speed-A1-type spiral CT; GE Healthcare, Pewaukee, WI, USA) was used to study the children. The scan parameters were as follows: tube voltage, 120 kVp; tube current, 100 mA; thickness, 5–10 mm; pitch, l; reconstruction matrix, 512×512; and scan mode, helical scan.

Certain children were subjected to brain MRI (Magnetom Aera 1.5T MRI; Siemens AG, Munich, Germany). The MRI examination was conducted approximately at the time of the patients’ hospitalisation. All of the MRI procedures used a 1.5T system. A conventional MRI sequence protocol was applied in all of the participants: spin-echo (SE) T1-weighted (T1-W) sagittal; SE dp/T2-W; fluid attenuation inversion recovery (FLAIR) axial; and turbo spin echo (TSE) T2-W and inversion-recovery (IR) coronal scans.

According to the imaging diagnostic standards of the brain and spinal cord injury, the congenital brain and skull deformities and PVL in children and infants were categorised into three degrees based on the damage in the periventricular region (8,9): i) for a mild degree, the white matter reduction was limited to the peritrigonal, anterior or posterior white matter; the central circle of the white matter appeared normal or slightly reduced, and the lateral ventricles were of normal size; the cerebral sulci were widened adjacent to the trigonal area of the lateral ventricle, and the lateral area exhibited a small visible area of softening, with long T1 and long T2 signals; ii) for a moderate degree, the white matter of the centrum semiovale was reduced, in addition to the previously described abnormalities; the optic radiation was involved; periventricular white matter was decreased significantly; deep lateral fissures were widened extensively with enlarged occipital horns located close to the lateral fissure with grey matter in close proximity to the walls of the lateral ventricles; the ependyma was separated from gray matter only by a small amount of white matter; the trigonal area was abnormally enlarged; and a small amount of malacia of the periventricular white matter was present. iii) for a severe degree, the cerebral white matter had almost disappeared; periventricular white matter was replaced with cystic areas and connected to the lateral ventricle; the lateral ventricle appeared irregular; the centre measured only half of the total residual amount of the white matter, grey matter and parietal grey matter, in which the lateral fissures were deep and close to the lateral ventricle. The group of patients with MRI was very small and not used for indexing purposes.

The reading, analysis and diagnosis of the imaging were conducted by two chief physicians of radiology and two chief physicians of children’s neurology and were blinded to other patient data. All radiology scans were read independently by two radiologists who were unaware of the clinical information. If these two radiologists disagreed in their analysis of the lesions, the films were sent to a third radiologist who was unaware of the opinions of the first two radiologists. Details regarding the procedure and efforts to minimise observer variability are presented in a previous study (10).

### Clinical diagnosis and evaluation

The diagnosis and evaluation involved the following. i) High risk factors of perinatal brain injury were obtained from information contained in a questionnaire that was completed by the mother of each child and provided to the doctors during the medical consultation. ii) CP was diagnosed, typed and classified according to the classification presented at an international symposium of cerebral palsy in 2006 (11). In this study, the following categories were considered for type according to clinical symptoms: spastic (diplegia, spastic quadriplegia, spastic hemiplegia, spastic monoplegia); flaccid; dyskinesia; ataxic; and mixed. iii) The children’s intelligence was evaluated according to the Gesell Developmental Scale and Wechsler Intelligence Scale. Children with a developmental quotient (DQ) of <75 or intelligence quotient (IQ) of <70 were diagnosed with mental retardation. iv) The brainstem auditory-evoked potential (MEB-9100 type-evoked potential) was used to assess the hearing threshold. Children with a hearing threshold >35 dB were diagnosed with hearing impairment. v) The sign-significant relations (S-S) (12) evaluation method developed by the China Rehabilitation Research Centre was used to diagnose significant language development delay. Children with a language assessment, language comprehension or expression delay of half a year or more compared with normal children of the same age were diagnosed with delayed language development. vi) Children with seizures were included as epilepsy cases and treated with antiepileptic drugs to control their symptoms. Epilepsy was diagnosed with reference to the diagnosis and classification criteria developed by the International League Against Epilepsy (13,14), and a 24-h dynamic abnormal EEG monitoring report was obtained for all children with epilepsy. vii) The children’s eyes were examined by the professional chief ophthalmologists in the hospital. Visually impaired patients were diagnosed to determine whether or not visual abnormalities, strabismus, amblyopia, eye involuntary movement, cortical blindness or nystagmus were present. The visual evoked potential and fundus examination were performed for non-responsive children with the application of strong light stimulation to the eyes.

### Statistical analysis

Data were stored on a computerised database and analysed using SPSS version 13.0 for Windows (Statistical Package for Scientific Studies for Windows; SPSS Inc., Chicago, IL, USA). The conditional logistic regression analysis method was used, in which PVL was considered as an independent variable, and the other factors were considered as dependent variables. Measurements are presented as the mean ± standard deviation and Wald tests were used in the statistical analysis. P<0.05 was considered to indicate a statistically significant result. Logistic regression analysis was performed using relevant factors.

## Results

### High risk factors

Among the 408 patients, 251 were born prematurely, 154 were born at full-term, and three were adopted and had an unknown gestational age. In addition, 80 cases had a gestational age of ≤28 weeks, with the lowest gestational age being 24 weeks. A total of 154 cases had a gestational age of ≥37 weeks. A total of 35 cases (8.58%) were born with a weight of ≤1,500 g, with the lowest weight being 900 g, and 181 cases (44.36%) were born with a weight of ≥2,500 g (Table I).

A total of 390 cases (95.59%; Table II) had one or more high risk factors, including 29 cases with simple prenatal risk factors, 94 cases with simple neonatal period risk factors and 263 cases with concomitant prenatal and neonatal risk factors. The three adopted children were born with an unknown history of birth or the mother’s pregnancy. Furthermore, 62 cases (15.20%) were twins or multiple births, including 7 cases with stillbirth, 9 cases with mortality after birth and 12 cases with CP. Among the 408 children, 181 cases were born with a birth weight of ≥2,500 g, considered a normal birth weight (44.36%), and these included 77 cases with neonatal asphyxia as one of the PVL risk factors (42.54%); only 12 cases had no risk factors (Tables III and IV).

### Logistic regression analysis

The analysis undertaken for gestational age, birth weight, single/multiple births, mode of delivery, amniotic fluid abnormalities, asphyxia, complications during pregnancy, encephalopathy and intracranial hemorrhage found a P-value<0.05 that indicated a statistically significant difference. Thus, these factors were associated with the occurence of PVL.

### CT findings and results

**Figure 1:**
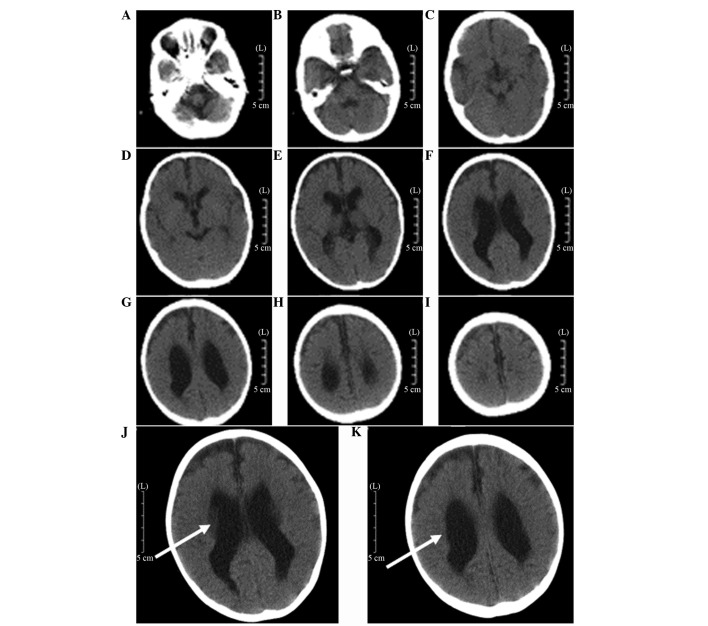
A 2-year old male premature infant with a gestational age of 34 weeks. (A–I) Computed tomography films subsequent to admission. In particular, (E–H) display the different degrees of the periventricular leukomalacia (PVL) manifestations and (F and G) are the more marked among these. (J and K) are amplification of figures F and G, respectively. The body of bilateral ventricles was largely expanded, the caudomedial part and occipital edges were not smooth, and the surrounding white matter was reduced (arrows).

The characteristics of PVL are generally consistent with the MRI findings (15,16). CT examination is not able to detect early damage in the periventricular white matter until cystic lesions are formed. The end-stage appearance of the PVL was observed by CT as follows (15): the lateral ventricle was enlarged, expanding from the rear of the lateral ventricles to the full lateral ventricle and the edges of the lateral ventricle were irregular and uneven. The quantity of the periventricular white matter was reduced mainly around the trigonal zone of the lateral ventricle and along the centrum semiovale in severe cases. The CT scan exhibited a low-density (Fig. 1). Analysis of the results indicated that 172 cases were classified as mild, 195 cases were moderate and 41 cases were severe (Table V).

### Association between PVL severity and gestational age, birth weight and type of CP

In this study, among the cases of severe PVL, 63.41% of the children had a gestational age of 24–28 weeks, 21.95% had a gestational age of 28–30 weeks and 97.56% with a gestational age of <37 weeks. Also, among the severe cases of PVL, 63.41% of children had a birth weight of 900–1,500 g, 26.83% had a birth weight of 1,500–2,000 g and 97.56% had a birth weight of <2,500 g. Spastic quadriplegia was present in 43.90% of the severe PVL cases. The results indicate that severe PVL was more common in infants born prematurely or with a low-birth weight. Hence, a young gestational age and low birth weight are associated with severe PVL. This result also indicated a high possibility of developing spastic quadriplegia in such cases (Tables V and VI).

The types of CP in the 408 children with PVL included the following: spastic CP in 390 cases (diplegia in 213 cases, quadriplegia in 114 cases, hemiplegia in 56 cases and monoplegia in 7 cases); 16 cases with hypotonia and 2 cases with dyskinetic CP. The incidences of other comorbidities were distinctly different among the different types of CP. The highest was hearing impairment in spastic diplegia. The total incidences of various comorbid disabilities with were higher in quadriplegia than in the other types (Tables VII and VIII).

## Discussion

CP is one of the most common pediatric neuromotor developmental disabilities with a prevalence rate of 2.37% (17). With the improvement of the conditions and technical advancements in perinatal medicine and neonatal emergency medicine, the survival rates of children born prematurely and other high-risk children have increased. The incidence of CP significantly increased by 66% to 100% (2). PVL has been defined as cyst formation with necrosis of myelinated fibres of the white matter in the trigonal region, situated at the posterior side and lateral to the external angles of the lateral ventricles in survivors of the perinatal asphyxia (18), with an incidence of 25–75% (19). PVL has a poor prognosis because of its unclear etiology and pathogenesis. As a major cause of early mortality, mental retardation and damage to the nervous system (20), PVL seriously affects the survival rates and the quality of life of preterm infants. Therefore, PVL has been the focus of numerous studies of the perinatal period. For instance, Drougia *et al* (4) demonstrated that PVL is the main cause of CP (particularly in children born prematurely), with ~50% of cases having CP and PVL. Considering that different onset types and comorbidities for CP affect the degree of disability and quality of life, the present study retrospectively analysed the images of 408 children with CP treated in the rehabilitation centre at a single hospital. The correlation between high risk factors (degree of PVL, gestational age, low birth weight and asphyxia) and CP, as well as clinical types and comorbidities, were investigated to evaluate the cause and clarify the underlying mechanism. The focus of the study was on perinatal period care, risk and early prevention, early diagnosis, early treatment and morbidity reduction.

Recent studies have indicated that PVL is associated with immature vascular development of the periventricular region, the imperfect autoregulation of cerebral blood flow and specific damage to oligodendrocyte precursors (21). Children born prematurely with immature brain development are very sensitive to hypoxia and ischaemia, and the periventricular area is the zone with the highest incidence of hypoxic-ischaemic damage in preterm infants. Therefore, damage of the periventricular white matter is more likely to occur in children born prematurely than in those born at full-term, and the degree is more severe. Perlman *et al* (22) observed that PVL mainly occurs in children born prematurely with a gestational age of <32 weeks and a birth weight of <1,500 g; the lower the birth weight, the greater the severity of the PVL. Vollmer *et al* (23) also found that the mortality rate of children born prematurely with a gestational age of <28 weeks was greater than that of those with a gestational age of 28–32 weeks after the PVL, and that such children usually succumb within 2 weeks after birth. Analysis of the 408 children with PVL in the present study indicates that 251 cases (61.51%) were born prematurely and 154 cases (37.74%) were born at full-term. A total of 35 cases (8.58%) had a weight at birth of ≤1,500 g, among which 26 cases had severe PVL, accounting for 63.41% of the total cases of severe PVL. A total of 226 cases (55.39%) were born with a weight ≤2500 g, of which 40 cases were severe PVL, accounting for 97.56% of the total cases of severe PVL. A total of 181 cases (44.36%) were born with a weight ≥2500 g, of which 1 case was severe PVL (0.24%). A total of 80 cases (19.61%) were born at a gestational age of ≤28 weeks, of which 26 cases were severe PVL (63.41% of all cases of severe PVL). A total of 150 cases (36.76%) were born at a gestational age ≤32 weeks, of which 38 cases were severe PVL (92.68%). Furthermore, 251 cases (61.52%) were born at a gestational age ≤37 weeks, of which 40 cases were severe PVL (accounting for 97.56% of all severe cases; Tables I, V and VI). The results indicate that the lower the gestational age and the lower the birth weight, the greater the severity of the PVL. This finding is roughly in accordance with the results of the studies previously mentioned. However, the proportion of the total cases was not very large, which is related to the objectives of the study, that the survival rate of children with more severe PVL is low, and that the lower the gestational age and the birth weight, the higher the incidence of the PVL.

In investigations of the etiology of PVL and the sequela CP, intrauterine infection has become topic of particular interest. According to epidemiological and animal studies, intrauterine infection/inflammatory response has an important role in the pathogenesis of PVL, as an alternative to hypoxia and ischaemia (24,25). Intrauterine infection can cause premature rupture of the membranes and preterm delivery (a high risk factor of PVL), and also directly causes fetal periventricular white matter damage (26). The incidence of neonatal PVL may be as high as 60–70% when intrauterine infection occurs in mothers, and more than half of the survivors have long-term neurobehavioral defects (27). A total of 154 children with CP and PVL in the present study had a normal gestational age, and mostly had PVL to a mild or moderate extent. Analysis of the high risk factors showed that infection, intrauterine hypoxia and other high risk factors were present in children with CP, suggesting that infection during pregnancy and intrauterine hypoxia are the main reasons for PVL in full-term infants.

Clinical studies have found that hypoxia and ischaemia in children and the degrees of asphyxia are closely associated with brain damage; wherein, the more severe the degree of the asphyxia caused by hypoxia, the more severe the brain damage (28). Other high risk factors, such as the mother suffering from pre-eclampsia, antepartum haemorrhage, premature rupture of membranes, fetal distress, intrauterine hypoxia and ischaemia and intrauterine infection (29), and the newborn baby suffering from seizures, mechanical ventilation, double fetal asphyxia, apnea, hypoxemia, hypocapnia and sepsis (27), easily cause changes in cerebral vascular and cerebral hemodynamics and damage to the autoregulation cerebral blood flow (15), resulting in hypoxic-ischaemic conditions in the brain (30), thereby leading to the occurrence of PVL. The results of the logistic regression analysis (Table III) showed that the high risk factors associated with PVL included gestational age, hypoxia, various complications during pregnancy, amniotic fluid abnormalities, intracranial hemorrhage, mode of delivery and single/multiple births. The correlation between birth weight and PVL was low, which was inconsistent with previous studies (31,32). The reason for this is hypothesised to be that the children in the present study were survivors of severe PVL, but the survival rate of very low birth weight infants with severe PVL is low. Encephalopathy was found to have a low correlation with PVL, suggesting that the occurrence of PVL is closely associated with the high risk factors of prenatal pregnancy; encephalopathy may be a secondary illness in these children. Reducing the birth rate of babies with a very low birth weight would decrease the occurrence of severe PVL and improve the neonatal survival rate.

The major clinical manifestations in children with PVL and CP were changes such as various degrees of movement disorders and mental retardation. Melhem *et al* found that the severity of motor impairment and cognitive impairment was closely associated with the lateral ventricular volumes, which is similar to the results of the present study (33). The movement disorders are considered to be associated with lesioning of the periventricular white matter affecting the pyramidal tract (34). The pyramidal tract starts from the cerebral cortex down to the anterior horn motor neurons of the spinal cord along the lateral ventricles. The order of distribution of nerve fibers from the pyramidal tract is lower limbs, trunk and upper extremities from the inside out. Therefore, based on the severity of PVL, lower limb disorders occur first, with clinical manifestations including spastic diplegia, and expansion of the lesions results in spastic quadriplegia (35). In the present study, 213 cases (52.21%) had spastic diplegia, and 114 cases (27.94%) had spastic quadriplegia. Spastic hemiplegia, spastic hemoplagia, hypotonia and dyskinesia accounted for 13.73, 1.72, 3.92 and 0.49% of cases, respectively. Imaging studies demonstrate the anatomical basis for the abnormal functioning of the brain (36). The clinical manifestations are associated with the severity of PVL in children. The neuromotor development of children with PVL can be predicted based on imaging findings. In the present study, 18 (43.90%) of the 41 children with severe PVL had spastic quadriplegia, whereas 13 cases (31.71%) had diplegia, and these were commonly found in children born prematurely or with a low birth weight. This finding suggests that the lower the gestational age and the lower the birth weight, the more severe the PVL, and the greater the scope of involvement in the corticospinal tract, the more likelihood the development of spastic quadriplegia and diplegia. The imaging of one of the children with dyskinesia showed severe PVL; however, the specific mechanisms remain unclear, but may be caused by concurrent pyramidal and extrapyramidal involvement (2).

In addition to movement disorders, the manifestations of PVL are often associated with epilepsy, visual and auditory damage, mental retardation and delayed language development. Optic radiation and auditory tract involvement in the wide ranging reduction of the periventricular white matter is the main cause of dysfunctions and damage of vision and hearing in children with PVL and CP. Epilepsy, hearing impairment and visual impairment were the three most common disorders. In the present study, the prevalence rate of vision and hearing impairment was 26.96%, in accordance with the literature. The prevalence of epilepsy was 7.35% (Table VIII), lower than the rate reported in the literature (5), which may be related to the fact that partial frequent seizures in children with CP were not rehabilitated and were missed. It has been suggested that the degree of lateral expansion and periventricular reduction of the white matter, thinning of the corpus callosum and optic radiation involvement are associated with IQ (37). In the present study, 17 children had various degrees of mental retardation. It has previously been shown that intellectual impairment is severe when the degree of lateral expansion and cortical damage increase with concurrent reduction of the periventricular white matter, indicating that PVL is an important clinical cause of mental retardation (38). In the present study, 16 children had different levels of language retardation. The language centres of Broca’s area, Wernicke’s area, the supramarginal gyrus and angular gyrus are all located in the periventricular area. When the range of the PVL incorporated the speech centre, the children would be likely to have various degrees of language retardation. The degree of audio-visual impairment, levels of intelligence, seizures and degree of language development can affect the therapeutic effect of CP, as well as the prognosis. Thus, vision and hearing examinations, intellectual measurement, language evaluation and EEG examination are recommended to be performed routinely in children with CP for early diagnosis and intervention, contributing to the improvement of the rehabilitation effect.

As one of the significant causes of CP in children, PVL is an ischaemic necrosis of the brain cells commonly found in infants born prematurely or with a low birth weight. The degree of PVL was negatively correlated to the gestational age and birth weight of the children. The degree of the PVL in full-term infants was associated with infection during pregnancy and intrauterine hypoxia. Quadriplegia and diplegia were most common among the subtypes of CP, and hearing impairment was the most common comorbidity. The type of CP most commonly associated with comorbidity was quadriplegia.

A retrospective clinical analysis with certain limitations was conducted in this study. However, this analysis was useful for evaluating the cause of the disease and understanding the correlation between the type of CP, comorbidities and the degree of PVL, and is a reminder that attention to perinatal care is important to avoid the birth of children with severe CP. The study suggested that improvement of the prognosis for children born prematurely or with a low birth weight with severe PVL may be achieved by observation and early intervention, and should be the direction of future research. The various pre-natal and post-natal risk factors affecting children with CP were strictly observed with the establishment of a collaborative network among the maternity, paediatrics and rehabilitation services. This was considered to be key for the prevention, diagnosis and treatment of children with CP, contributing to enhancement of the quality of the population, reduced morbidity rates and improved life quality of children with CP.

## Figures and Tables

**Table I:** Gestational age and birth weight in children with PVL.

Variable	No. of cases (%)
Gestational age (weeks)	
24–28	80 (19.61)
28–30	12 (2.94)
30–32	58 (14.22)
32–34	29 (7.11)
34–37	72 (17.65)
37–41.6	154 (37.75)
Unknown^a^	3 (0.74)
Birth weight (g)	
900–1500	35 (8.58)
1500–2000	111 (27.21)
2000–2500	80 (19.61)
2500–5500	181 (44.36)
Unknown	1 (0.24)

PVL, periventricular leukomalacia.

a Adopted children with unknown gestational age.

**Table II:** High risk factors in children with PVL.

Factors	No. of cases (%)
Prenatal	
Abnormal amniotic fluid	54 (13.24)
Umbilical cord abnormalities	16 (3.92)
Placental abnormalities	29 (7.11)
Intrauterine distress	27 (6.62)
Threatened abortion	57 (13.97)
Pregnancy-induced hypertension	26 (6.37)
Pre-eclampsia	1 (0.25)
Infection during pregnancy	86 (21.08)
TORCH infection	175 (42.89)
Habitual abortion	7 (1.72)
Gestational diabetes	9 (2.21)
Intrapartum and postnatal	
Caesarean section	208 (50.98)
Twins/multiple births	62 (15.20)
Neonatal asphyxia	181 (44.36)
Neonatal encephalopathy	123 (30.15)
Neonatal sepsis	5 (1.23)
Neonatal respiratory distress	11 (2.69)
Neonatal seizures	18 (4.41)
Neonatal mechanical ventilation	9 (2.21)
Neonatal intracranial haemorrhage	37 (9.07)
Neonatal jaundice	169 (41.42)
Neonatal pneumonia	25 (6.13)

TORCH, toxoplasmosis, other agents, rubella, cytomegalovirus or herpes simplex.

**Table III:** Logistic regression analysis of high risk factors for PVL.

							95% CI for EXP (B)	
							
Variables^a^	B	S.E.	Wald	df.	Sig	Exp (B)	Lower	Upper
Gestational age	0.384	0.089	18.763	1	<0.001	1.468	1.234	1.747
Birth weight	−0.181	0.133	1.870	1	0.172	0.834	0.643	1.082
Single/multiple births	0.843	0.282	8.927	1	0.003	2.324	1.337	4.040
Mode of delivery	0.893	0.168	28.255	1	<0.001	2.442	1.757	3.393
Amniotic fluid abnormalities	1.459	0.355	16.927	1	<0.001	4.304	2.147	8.625
Asphyxia	1.381	0.210	43.209	1	<0.001	3.980	2.636	6.008
Complications during pregnancy	1.613	0.205	62.078	1	<0.001	5.015	3.358	7.490
Encephalopathy	0.045	0.226	0.040	1	0.841	1.046	0.671	1.631
Intracranial hemorrhage	2.338	0.412	32.199	1	<0.001	10.358	4.620	23.226
Constant	−3.561	0.393	82.071	1	<0.001	0.028		

a Variable(s) entered in step 1.

PVL, periventricular leukomalacia. B is the coefficient of the independent variable; SE is the standard differential; Wald is the Wald statistic; df, degrees of freedom; Sig indicates the P-value; Exp (B) indicates the odds ratio (OR); 95% CI for EXP (B) indicates the 95% confidence interval of the OR value.

**Table IV:** High risk factors in 181 children with normal birth weight and PVL.

Factors	Cases (%)
Prenatal	
Abnormal amniotic fluid	30 (16.57)
Umbilical cord abnormalities	15 (8.29)
Placental abnormalities	11 (6.08)
Intrauterine distress	12 (6.63)
Threatened abortion	22 (12.15)
Pregnancy-induced hypertension	6 (3.31)
Pre-eclampsia	1 (0.55)
Infection during pregnancy	49 (27.07)
TORCH infection	82 (45.30)
Neonatal pneumonia	11 (6.07)
Intrapartum and postnatal	
Cesarean section	91 (50.27)
Twins/multiple births	15 (8.28)
Neonatal asphyxia	77 (42.54)
Neonatal encephalopathy	52 (28.73)
Neonatal sepsis	4 (2.21)
Neonatal respiratory distress	3 (1.66)
Neonatal seizures	12 (6.63)
Neonatal mechanical ventilation	4 (2.21)
Neonatal intracranial hemorrhage	11 (6.08)
Neonatal jaundice	76 (41.99)

TORCH, toxoplasmosis, other agents, rubella, cytomegalovirus or herpes simplex.

**Table V:** Comparison of gestational age with the degree of PVL [n (%)].

	Degree of PVL		
	
Gestational age (weeks)	Mild	Moderate	Severe
24–28	14 (3.43)	40 (9.80)	26 (6.37)
28–30	1 (0.25)	2 (0.49)	9 (2.21)
30–32	16 (3.92)	39 (9.56)	3 (0.74)
32–34	10 (2.45)	18 (4.41)	1 (0.25)
34–37	41 (10.05)	30 (7.35)	1 (0.25)
37–41.6	89 (21.81)	64 (15.69)	1 (0.25)
Unknown^a^	1 (0.25)	2 (0.49)	0
Total	172	195	41

PVL, periventricular leukomalacia.

a Adopted children with unknown gestational and birth history.

**Table VI:** Comparison of birth weight and the degree of PVL [n(%)].

	Degree of PVL		
	
Birth weight (g)	Mild	Moderate	Severe
900–1500	3 (0.74)	6 (1.47)	26 (6.37)
1500–2000	30 (7.35)	70 (17.16)	11 (2.70)
2000–2500	36 (8.82)	41 (10.05)	3 (0.74)
2500–5500	103 (25.25)	77 (18.87)	1 (0.25)
Unclear	0	1 (0.25)	0
Total	172	195	41

PVL, periventricular leukomalacia.

**Table VII:** Comparison of cerebral palsy type and the degree of PVL (no. of cases).

	Degree of PVL		
	
Type of cerebral palsy	Mild	Moderate	Severe
Diplegia	97	103	13
Spastic quadriplegia	45	51	18
Spastic hemiplegia	21	30	5
Spastic monoplegia	6	1	0
Hypotonia	3	9	4
Dyskinesia	0	1	1
Total	172	195	41

PVL, periventricular leukomalacia.

**Table VIII:** Comparison of the type of cerebral palsy and comorbidities in children with PVL (no. of cases).

Type of cerebral palsy	Cases	Visual impairment	Hearing impairment	Language barriers	Mental retardation	Epilepsy
Diplegia	213	7	47	8	4	3
Spastic quadriplegia	114	10	30	4	10	26
Spastic hemiplegia	56	2	4	2	2	1
Spastic monoplegia	7	1	2	0	0	0
Hypotonia	16	1	6	0	1	0
Dyskinesia	2	0	0	2	0	0

PVL, periventricular leukomalacia.
